# Usefulness of Photodynamic Therapy as a Possible Therapeutic Alternative in the Treatment of Basal Cell Carcinoma

**DOI:** 10.3390/ijms161023300

**Published:** 2015-09-28

**Authors:** Paola Savoia, Tommaso Deboli, Alberto Previgliano, Paolo Broganelli

**Affiliations:** 1 Department of Medical Sciences, University of Turin, Torino 10126, Italy; E-Mails: tommaso.deboli@alice.it (T.D.); alberto.previgliano@unito.it (A.P.); 2 Città della Salute e della Scienza, Turin 10126, Italy; E-Mail: paolobroganelli@inwind.it

**Keywords:** photodynamic therapy, PDT, basal cell carcinoma treatment, non-melanoma skin cancer

## Abstract

Basal cell carcinoma (BCC) is the most common cancer in individuals with fair skin type (I–II) and steadily increasing in incidence (70% of skin malignancy). It is locally invasive but metastasis is usually very rare, with an estimated incidence of 0.0028%–0.55%. Conventional therapy is surgery, especially for the H region of the face and infiltrative lesions; in case of inoperable tumors, radiotherapy is a valid option. Recently, topical photodynamic therapy (PDT) has become an effective treatment in the management of superficial and small nodular BCC. PDT is a minimally invasive procedure that involves the administration of a photo-sensibilizing agent followed by irradiation at a pre-defined wavelength; this determines the creation of reactive oxygen species that specifically destroy target cells. The only major side effect is pain, reported by some patients during the irradiation. The high cure rate and excellent cosmetic outcome requires considering this possibility for the management of patients with both sporadic and hereditary BCC. In this article, an extensive review of the recent literature was made, in order to clarify the role of PDT as a possible alternative therapeutic option in the treatment of BCC.

## 1. Introduction

Basal cell carcinoma (BCC) is a very common malignancy, accounting for 80% of non-melanoma skin cancers (NMSCs) and affecting around two million people each year, with a steadily increasing incidence [1,2]. It develops mainly in subjects with fair skin and a history of chronic sun exposure, but certain categories of patients, such as solid organ transplant recipients and/or chronically immunosuppressed people, are most frequently affected. In addition, other risk factors include scars, exposure to arsenic, and hereditary disorders such as nevoid basal cell carcinoma syndrome (Gorlin-Goltz syndrome) and xeroderma pigmentosum [3]. The site of predilection is represented by chronically sun-exposed skin of the head and neck region, but multicentre superficial BCC frequently arise on the trunk. The median age at onset is over 60 years, even if diagnoses made in people younger than 40 years are progressively increasing [1]. Metastases from BCC are very rare, with an estimated incidence of 0.0028%–0.55% [4]. However, the tumor is locally invasive and can infiltrate and destroy the subcutaneous tissue, bone and cartilage, reaching vital structures (major vessels or central nervous system); complications like bleeding and infections can affect the prognosis of patients, especially when elderly and debilitated [2].

Currently, multiple therapeutic options are available for the treatment of BCC, providing an excellent response rates in most of cases treated. However, the fact that the diagnosis of BCC is frequently carried out on patients with multiple comorbidities makes alternative minimally invasive procedures necessary.

Photodynamic topical therapy (PDT) is a procedure based on the administration of a photo-sensibilizing agent, followed by irradiation at a pre-defined wavelength [5]. The creation of reactive oxygen species specifically destroys neoplastic cells, representing an effective treatment for certain types of skin cancers [6]. PDT is generally well tolerated, with minimal side effects, and this therapeutic option should be take into consideration for the treatment of BCC, if patients are properly selected [5,6].

This paper, starting from an extensive review of the literature related to the topic, describes the main treatment options currently available for the management of BCC, with particular attention to recent results on the use of PDT in this disease and on its potential benefits and possible limitations.

## 2. Clinical and Histopathological Aspects of BCC

### 2.1. Clinical Features

BCC usually occurs as a single lesion, even if the appearance of simultaneous or subsequent lesions is a not infrequent event.

Five main clinic types of BCC has been described [7,8,9,10,11,12,13]:

- Nodule-ulcerative BCC is the most common variant, accounting for almost 80% of cases and is represented by a small nodule with teleangectasic vessels on his surface [7,8,9]. The nodule progressively increases in size and often develops a central ulceration, surrounded by a pearly border; however, nodular BCCs are typically indolent and slow growing.
- Invasive BCC (*basalioma terebrans*) is a rare variant that growth aggressively and can invade deep tissues and bone, destroying progressively structures as the nose or eyes [10].
- Pigmented BCC shows clinical features similar to nodule-ulcerative BCC, but differs for the presence of brown or dark pigmentation.
- Morpea-like or fibrosing BCC account for almost 6% of BCC and occurs mainly on the face; is represented by a flat or slightly depressed indurate plaque, with ill-defined borders and shows a more aggressive history, with frequent recurrences [7,8,9,11].
- Superficial BCC account for up to 15% of cases and consists of a erythematous, slightly infiltrated patch, mainly located on the trunk, with a tendency to increase by a peripheral extension. Often there are a fine pearly border around the patch and small ulcerations on its surface [7,9,12,13].

### 2.2. Histopathological Features

BCC is characterized by the presence of cells with a large, hyperchromic nucleus and scanty cytoplasm. The majority of BCCs are well demarcated and peripheral cells shows a palisade arrangement; however, areas of infiltrative growth can be present and fibrotic changes in the dermis could be a marker for more aggressive variants [14].

The stromal tissue is arranged in parallel bundles around the tumor masses and frequently there are areas of stromal retraction from the tumor islands, which result in peritumoral lacunae. The typical stroma of BCC is fibromyxoid, with presence of mucin, rare infiltrating lymphocytes and higher number of dilated vessels. These characteristics represent a useful diagnostic clue; moreover, the presence of stroma at the margin of the lesion suggests an incomplete tumor removal and represents an indication for re-excision [15]. Quantitative differences in the peritumoral stroma among BCC subtypes have also been demonstrated [16]. BCC is highly dependent to the surrounding stroma for survival; nonspecific inflammatory phenomena can disrupt this stroma and play an important role in tumor involvement, explaining the spontaneous or biopsy-induced regression of BCC that is occasionally reported [17].

As previously demonstrated by Roozeboom *et al.* [18], in BCCs with a maximum tumor thickness of 1.0 mm, histologic characteristics, tumor thickness and adnexal extension are not associated with treatment failure. However, tumour biopsy is recommended in order to avoid a clinical underestimation of the BCC thickness that may result in a treatment failure [19].

Conversely, the Fiechter’s retrospective histopathological analysis [20] of BCCs recurring after methyl aminolevulinate (MAL)-PDT provides evidence that 62.5% of the superficial or nodular primary BCCs show a relapse characterized by a more aggressive histological pattern. Although the latter may reflect the natural course of tumor, their data suggest that BCCs recurring after PDT show an as yet unrecognized risk of behaving more aggressively [21]. Some BCC cells that have received a sublethal dose of PDT could accumulate enough DNA damage to behave more aggressively.

## 3. Conventional Treatment of Localized BCC

Conventional therapies for the treatment of the BCC are many, and the choice between different options should take into account both age and comorbidities affecting the patient and clinical and histopathological features of the lesion to be treated [22]. Superficial and nodular BCC are usually considered at lower risk of recurrence, whereas this is increased for infiltrative and micronodular subtypes. Lesions that develop in sensitive anatomic areas, such as face, extremities and genitalia and those measuring more than 2 cm in diameter are classified as high-risk [23,24,25]. Indeed, BCCs can be classified as low- or high-risk lesions also on the basis of others factors, including the presence of well-defined *vs.* ill-defined borders, the presence or absence of perineural invasion, recurrent *vs.* primary disease, previous radiotherapy and immunosuppression status [22].

### 3.1. Surgery

The first treatment choice for BCC management is represented by surgery, given a relatively low rate of recurrence and the possibility to have a histological control of the tumor. However, there are several disadvantages, represented by the usual operative risks, aesthetically or functionally disturbing scars and pigment changes, as well as possible delays in wound healing [26]. The goal of the treatment is represented by the tumor eradication with the minimal destruction of the tissue in order to preserve the function and the appearance. So, the choice between the different surgical options should be determined on the basis of the possible presence of risk factors in the tumor to be treated.

Curettage with electrodessication, and surgical excision with postoperative margins control are considered the first-rate treatments for low-risk BCCs [22], giving a five-year overall cure rate of 92% and 98%, respectively [27,28,29]. However, Mohs surgery is the treatment of choice in all high-risk lesions and for BCC localized on eyes, lips or nose, with a five-year disease free survival rate of 99% [2,26,30].

### 3.2. Liquid Nitrogen Cryotherapy and Ablative Laser Therapy

Cryotherapy obtains destruction of the neoplastic tissue by low temperature freezing. The rationale is based on the fact that the high water content, high metabolism and high number of blood vessels make neoplastic cells more sensitive to low temperature [31]. Moreover, the benefits of this therapy include its ability to trigger a specific T lymphocyte response and to increase the activity of natural killer cells by releasing tumor antigens and inflammatory mediators.

Cryotherapy is simple and money saving and is commonly applied to the treatment of NMSC precursors. However, it can also be occasionally used in superficial or small-size BCCs [26,31]; two randomized controlled trials showed cure rates ranging from 85% to 95% [32,33]. Side effects develop in 20%–50% of patients and include pain, erythema, blisters and crusts. The main disadvantages of this procedure are represented by hypopigmentation or scarring, and by the lack of histological control [32]. Similar problems are observed with ablative laser treatment, that is usually performed with CO_2_ or Erbium YAG laser.

### 3.3. Topical Treatment Strategies

The cytostatic agent 5-fluorouracil (5-FU) is available as a 5% cream that is designed for twice daily application for 3–12 weeks until erosions develop. The 5-FU is a chemotherapic agent that interferes with DNA synthesis by inhibiting thymidylate synthetase, thus decreasing cell proliferation; as a final effect cellular apoptosis is obtained. The experience on the use of 5-FU to treat BCC is well established. In fact, since the seventies, several studies [34,35,36] demonstrated the effectiveness of 5-FU in more than 80% of superficial BCCs. A more recent study on 31 BCCs demonstrated that the use of twice-daily 5-FU for an average of 11 weeks might lead to a 90% histologic cure rate [37]. A large multicenter single-blinded randomized comparative trial [38] reported instead a cure rate of 80%. Advantages of topical 5-fluorouracil are scar-free healing and the possibility to treat wider areas. Disadvantages are the sometimes severe inflammatory and erosive reactions, which develop at individually variable time intervals (days to weeks) and the dependence on good patient compliance.

Topical imiquimod binds the toll-like receptor 7 and provides immunomodulatory effects by increasing the release of pro-inflammatory cytokines; it is currently approved for the treatment of small superficial BCC, with a complete cure rate around the 80% [39,40]. Due to the fact that tumour thickness is considered as a predictor of response in patients treated with imiquimod [41,42,43], reports about successful treatment of large BCC are rare. Indeed, few cases of giant tumors cured without pathological evidence of recurrences after a long-term follow-up have been reported [44]. Reported adverse events are erythema, flaking, blistering, erosions and ulceration; most of them resolve after eight weeks [45].

Recently, the Food and Drug Administration (FDA) approved ingenol mebutate (the active principle extracted from the sap of *Euphorbia peplus*) [46] for the treatment of precancerous lesions. Few cases of home treatment of NMSC with the sap of *Euphorbia peplus* are reported by literature [46,47] and experimental studies suggested a possible role of ingenol mebutate also for BCC patients who failed or were ineligible for conventional treatments [48,49]. For patients affected by BCC, the complete clinical response after one month was 82%, whereas after a mean follow-up of 15 months, the response rate was 57%. In another phase II trial [48], a histologically confirmed clearance of the treated lesions occurred in 65% of the patients; efficacy appeared to be dose-dependent and significantly higher in patients treated for two consecutive days.

### 3.4. Radiotherapy

Radiotherapy may be considered when the tumor site or size prevents surgical approach, or when the patient's medical history contraindicates other types of treatment. Other indications are represented by incomplete surgical excision or relapses, when re-intervention is not technically feasible [26]. The S2-K guidelines recommend high-energy radiation therapy (electron or photon beam); primary tumors are treated with daily doses of 2–3 Gy and a total dose of 60–70 Gy. After R1 resection, 50–60 Gy are recommended and after R2 resection, 60–70 Gy [2]. Large tumors or those in unfavorable locations can be treated by intensity-modulated radiation therapy or tomotherapy [50,51].

Large retrospective studies reported a five-year overall response varying between 88.6% and 96.1%, according to the size of the neoplasia [26,52,53].

## 4. PDT in the Treatment of Localized BCC

### 4.1. Background

PDT is a clinically approved, minimally invasive therapeutic procedure with a selective cytotoxic activity, which is successfully used for the management of neoplastic and non-malignant diseases [5].

PDT requires three essential components: a photosensitizer, a light source, and oxygen. When the photosensitizer is exposed to specific wavelengths, it becomes active from a “ground state” to an “excited state”; as it returns to the ground state, the energy released can mediate selective cell killing [6]. The selectivity of the photosensitizer uptake and the duration and depth of the light source minimize the damage to surrounding healthy tissue; in fact, PDT has a low potential for causing DNA damage, mutation, or carcinogenesis [54].

There are many types of photosensitizers available and several routes (topical, oral, or intravenous) by which they can be delivered to the patient. Currently, the only photosensitizers approved for dermatologic indications are aminolevulinic acid (ALA) and methyl aminolevulinate (MAL); both are prodrugs that require conversion to photo-activable porphyrin [55]. ALA crosses the cell membrane, and conversion to prothoporpirin IX (PpIX) occurs inside the cell. However ALA-esters, such as MAL, are first hydrolyzed to ALA in the cytosol [56].

Additional factors also affect the efficiency of PpIX production and higher temperatures facilitate conversion [57].

ALA has low lipid solubility, limiting its ability to penetrate through skin (no deeper than 2 mm) or cell membranes and thus restricting its use in PDT to superficial diseases [58]. MAL is reported to have increased lipophilicity and deeper skin penetration when compared with ALA [59].

*In vivo* and *ex vivo* imaging results demonstrate the potential of Vitamin D as a combination treatment to enhance ALA induced PpIX synthesis, resulting in higher tumor cell death following PDT [60].

### 4.2. Patient Selection and Technical Aspects

PDT is generally recommended as a treatment option for superficial and thin nodular BCC (thickness < 2 mm). However, some investigators accept 2–3 mm thick BCC for PDT when combined with a previous lesion curettage. In fact, the main barrier to the absorption of photosensitizers is the stratum corneum [61] and preparatory steps are necessary to improve photosensitizer uptake [62]. Some practitioners have observed reduced efficacy if lesions are not debrided prior to PDT [63].

Occlusion of lesions with a keratolytic the night before treatment can facilitate crust removal. Tape-stripping, microdermabrasion, laser ablation or gentle curettage can also be used to reduce hyperkeratosis [57,62,63,64,65,66,67].

Christensen *et al.* [18] demonstrated that the mean reduction of tumor thickness after deep curettage was 50% and was independent from the histological subtype. So, even if curettage before topical PDT may significantly improve complete clearance rate, results obtained in nodular subtypes remain scanty.

Treatment of scalp lesions need to be particularly careful, and might also be more difficult due to the presence of hair. Cutting hair before treatment can be helpful to localize the extension of the lesions.

### 4.3. Clinical Results

#### 4.3.1. ALA-PDT in Treatment of BCC

The main studies recently published about the use of ALA-PDT in treatment of BCC are reported in Table 1.

A 10-year longitudinal study performed on 60 lesions found that the complete response (CR) rate for primary lesions after superficial curettage was 78%, with 63% after one session and 90% after two sessions. This study used ALA-PDT with halogen light (light intensity 150–230 mW/cm^2^). The cosmetics outcome was rated as good or excellent in over 91% of the evaluated cases [68]. Another study (*n* = 94) compared the CR rate (mean follow-up 25 months) for sBCC in patients who received ALA-PDT (31/31, 100%) *vs.* surgery (28/29, 96.55%). Patients with nBCC had better response rates with surgery (16/17, 94.12%; *p* = 0.88) than those treated with ALA-PDT (15/17, 88.24%) [69]. Wang *et al.* [70] demonstrated that the efficacy of ALA-PDT is similar to that of cryosurgery, with the advantage of greater cosmetic results, achieved in a lesser healing interval.

A study published in 2012 by Osiecka and colleagues [71] evaluates the use of combined therapy encompassing both ALA-PDT and imiquimod 5% on a group of 34 patients that were randomized between ALA-PDT combined with vehicle placebo cream (*n* = 10 patients) and ALA-PDT combined with topical applications of imiquimod (*n* = 24 patients); the authors encountered a complete remission in 60% of the patients from the first group, compared to 75% in the second group [71].

ALA-PDT does not seem to be an efficient option for the treatment of nodular forms. In the largest single institution experience with 1440 nodular and superficial BCCs, PDT shows an initial CR rate of 92%. However, the response rate dropped to 71% in 75 patients with nodular BCC [72]. Moreover, a controlled randomized study enrolling 149 patients with 173 BCCs, surgically excised with safety margins of 3 mm or treated with ALA-PDT, proved a 3-years recurrence rate of 2.3% for the surgical excision, compared to 30.3% for PDT [73].

**Table 1:** Clinical results of ALA-PDT in treatment of BCC.

Authors	No. of Treated Lesions	Histotype	Treatment Outcome	Follow-up (Months)
Zeitouni *et al.*, 2001 [72]	1440	sBCC/nBCC	CR 92%	6
	75	nBCC	CR 71%	NA
Mosterd *et al*., 2008 [73]	173 (149 pts)	BCC	CR 69.7%	36
Christensen *et al*., 2012 [68]	60	BCC	CR 78%	120
Osiecka *et al*., 2012 [71]	34	BCC	CR 60% (ALA-PDT + placebo)	
			CR 75% (ALA-PDT + imiquimod)	
Cosgarea *et al*., 2013 [69]	94	sBCC	CR 100%	25
		nBCC	CR 88.24%	

CR: complete remission; NA: not available.

#### 4.3.2. MAL-PDT in the Treatment of BCC

MAL-PDT is a very safe and efficient therapeutic approach, mainly for patients with superficial variants of BCC, as demonstrated in the majority of published series. The main studies recently published about this topic are reported in Table 2. In 2001, Soler *et al.* [74] published a study on 350 superficial BCCs and previously curetted nodular BCCs, on which 160 mg/g concentration MAL was applied 3 h before illumination with a PDT lamp. At the end of the study, 310 of the 350 treated lesions remitted completely, 89% of them maintaining the remission even after three years. The cosmetic results were reported as good or excellent in 98% of the cases that achieved complete remission [74]. In another study published in 2011 [75] Fantini *et al.* reported an overall response rate of 62%, varying from 82% for sBCC and 33% for nBCCs. The study proved that both limb location of BCCs and the presence of ulceration negatively affect the efficacy of MAL-PDT whereas the size of the lesions does not seem to influence the outcome of the procedure.

Also for MAL-PDT, the efficacy of the technique is inversely related to the thickening of the lesion, as consequence of the physical ability of the luminous radiation to penetrate the skin. A study conducted on 95 BCCs of different thickness, stratified the results depending on the thickness of the lesion, as it follows: CR in lesions with thickness less than 1.3 mm, partial response in lesions between 1.3 and 1.8 mm and lack of response in those that exceeded 1.8 mm in thickness, with a two-year overall response rate of 75.8% [76].

Two randomized, multicenter, double-blind trials performed in Australia support the efficiency of MAL-PDT in the treatment of nodular BCC. The trials enrolled 131 patients, 66 of whom were referred for MAL-PDT treatment and 65 were treated with placebo-PDT. A histologically confirmed complete remission was observed in 75% of patient treated with MAL-PDT and only in 27% in the group treated with placebo-PDT [77]. MAL-PDT is reported also as adjunctive therapy for sclerodermiform BCC prior to standard surgical excision in order to reduce tumor size [78] and may be also considered for pigmented BCCs after an adequate debulking; however, patients should be closely followed due to higher recurrence risks [79].

MAL-PDT is also efficient for BCCs that are considered “difficult to treat” in respect to their dimensions or topographic location or to patient general clinical condition. An open, prospective, uncontrolled study assessed 94 patients with 123 “difficult to treat” BCC, with a remission rate of 92% for superficial BCC and 87% for the nodular ones [80]. Similar encouraging results were obtained also in the treatment of BCC of the eyelid margin [81,82]. On the contrary, lesions in the H-zone have reduced clearance rates.

**Table 2:** Clinical results of MAL-PDT in treatment of BCC.

Authors	No. of Treated Lesions	Hystotype	Treatment Outcome	Follow-up (Months)
Soler *et al.*, 2001 [74]	350	sBCC/nBCC	CR 88.57%	36
Horn *et al.*, 2003 [80]	123 (94 pts)	sBCCs	CR 92%	24
		nBCCs	CR 87%	
Foley *et al.*, 2006 [77]	131	BCC	CR 75% (MAL-PDT)	NA
			CR 27% (PDT + placebo)	
Eibenschutz *et al*., 2008 [83]	14 < 50 mm	BCC	CR 95%	6
	40–49 mm		CR 66%	36
Li *et al*., 2010 [76]	95 (47 pts)	sBCC	OR 75.8%	24
Fantini *et al*., 2011 [75]	194 (135 pts)		OR 62%	
		sBCC	CR 82%	NA
		nBCC	CR 33%	

CR: complete remission; OR: overall response; NA: not available.

MAL-PDT also proves good results in treating giant BCC. Eibenschutz *et al.* [83] presented a series of giant and large BCC, showing a 95% remission rate after six months; three years post-therapy, the remission rate was maintained at 66% of the treated cases. The association of MAL-PDT with topical imiquimod leads to a considerable reduction in the tumoral dimensions, allowing a subsequent excision to be performed in safe conditions [84].

### 4.4. Side Effects and Follow-up

Throughout, PDT is a well tolerated and a relatively cost-sparing technique. Absolute contraindications to PDT include history of porphyria, systemic lupus erythematosus or other photosensitive dermatoses, and allergy to the active ingredients in the photosensitizer, which is considerably rare.

Common side effects of this technique include erythema, edema, itching, epithelial exfoliation, pustules, and post-inflammatory hyperpigmentation, especially for Fitzpatrick skin phototype IV–V [85]. The most common reaction is represented by pain during administration [86], especially in patients treated with ALA, as demonstrated in comparative studies with MAL-PDT [87].

The recommended PDT treatment schedule for BCC in the majority of European countries is represented by two session at baseline, one week apart, with a re-evaluation after three months and a second cycle if necessary. In doubtful cases, the clinical evaluation may be supported by a biopsy to confirm the successful remission of the lesion; till now only few reports are available concerning the utility of dermoscopy in the assessment of lesions after PDT or other non-ablative therapies [88].

## 5. The “Nevoid Basal Cell Syndrome” (Gorlin-Goltz Syndrome)

### 5.1. Clinical Features

The nevoid basal cell syndrome is an autosomal dominant disorder with a low penetration that was described for the first time by Gorlin and Goltz in 1960 [89,90]. In Europe, the approximate prevalence of this syndrome is reported to be 1 in 30,827 (England) to 1 in 256,000 (Italy). More recently, the gene responsible for Gorlin-Goltz syndrome has been identified as PTCH1, which encodes the receptor for Sonic hedgehog protein [91,92]. Because the hedgehog-signaling pathway plays a pivotal role in embryonic development and tumor genesis, its deregulation results in various developmental defects and tumors. The patients affected by Gorlin-Goltz syndrome suffer since puberty with hundreds of small BCCs that increase progressively in number and size and are distributed randomly over the entire face or body. During adulthood, tumors became progressively invasive and can cause significant tissue destruction [93]. Patients affected by Gorlin-Goltz syndrome often show numerous palmo-plantar pits, epidermal cysts and different skeletal and central nervous system abnormalities. Moreover, they are more prone to develop various tumors, as medulloblastoma, ovary carcinoma, cardiac fibroma and odontogenic tumors [90].

### 5.2. Treatment

No guidelines exist for the treatment of Gorlin-Goltz syndrome. Since this condition is chronic and patients will continue to develop new lesions throughout their life, the goal of treatment is not necessarily complete remission [94]. Radiotherapy is contraindicated in these patients because it may induce new BCCs [95]. Lesion response rate to imiquimod, 5-FU and cryotherapy for sBCC in Gorlin-Goltz is variable.

Gorlin-Goltz patients have numerous lesions, so it is preferable to treat an entire area with field therapy rather than a single lesion approach; MAL-PDT can be considered and may lead to a potential reduction in surgical procedures. Although MAL-PDT is not approved for children, pediatric patients with Gorlin-Goltz might also be evaluated for this therapy. A recent case report also suggested a chemo preventive role of PDT in a patient with Gorlin-Goltz syndrome [96].

## 6. Treatment of Advanced BCC

Until a few years ago, the therapeutic approach for patients with locally advanced or metastatic NMSCs was exclusively represented by conventional chemotherapy, based on platinum, taxanes, or combinations of 13 *cis*-retinoic acid and interferon-alfa [35]. The remission rate reached by these therapies was low and serious side effects were frequent.

The above-mentioned recent identification of PTCH1 mutations in BCC led to the development of the hedgehog (SMO) inhibitor vismodegib, that demonstrated a more specific antitumor activity and less toxicity than conventional drugs [97] and was approved by the FDA in January 2012 and by the European Medicines Agency (EMA) in July 2013. The administration of vismodegib 150 mg daily showed a response rate of 58% with a median duration of 12.8 months in a Phase I study involving 33 patients with advanced BCCs [98]. These encouraging results were confirmed by other multicentric studies that enrolled patients with advanced, inoperable or metastatic BCCs [97,99]. Preliminary promising data were also reported for the prevention and treatment of BCCs in patients with the basal cell nevus syndrome but a Phase III study is still ongoing.

Hedgehog pathway inhibitors are markedly teratogenic and embryotoxic, on the other hand vismodegib is quite-well tolerated, with specific mechanism-related side effects, including muscle spasms, alopecia, dysgeusia, weight loss, and fatigue [97]. However, a crucial point that needs further investigations is represented by the development of resistance to vismodegib during long treatments, probably due to the onset of new mutations in SMO [98].

Recently, the Committee for Medicinal Products for Human Use (CHMP) has given a favorable opinion to sonidegib, and several studies about other new SMO inhibitors are ongoing [97,98,99] both on advanced BCC and on other solid tumors.

## 7. Future Perspectives in BCC Treatment

Daylight photodynamic therapy (DL-PDT) is a promising treatment for multiple grade I and II actinic keratosis on the face and scalp; this type of treatment was approved in 2014 in Australia, Brazil and Colombia but not yet in European countries [100]. Daylight photodynamic therapy using MAL has also been used in an exploratory study of 21 patients with 32 BCCs who received two treatments one week apart, achieving a 90% three month complete clinical response rate; however six patients recurred during a 12 months follow-up [101].

Nanotechnology has been explored as a novel platform for cancer treatment, with a potential role also in skin tumors, even if today data about its specific application to BCC are not available. Nanocarriers for drugs have the potential to enhance the therapeutic efficacy of a drug, since they can be engineered to modulate its release and stability, and to prolong its circulation time, protecting it from elimination by phagocytic cells or premature degradation. Moreover, nanoscale carriers can be tailored to accumulate in tumor cells and tissues, both through the enhanced permeability and retention effect, and by active targeting strategies using ligands designed to recognize tumor-associated molecular markers [102,103].

Nanoparticles may be used as a base for the construction of multifunctional nanoscale devices. These devices offer the opportunity to combine diagnostic, imaging or targeting agents with therapeutic agents in the same package. Thus far, several kinds of nanoparticles have been engineered and used in PDT applications; these nanoparticle agents range from liposomes, oil-based dispersions, polymeric particles and hydrophilic polymer–photosensitizer conjugates to gold nanoparticles. The porous structure of silica nanoparticles can not only act as a suitable carrier for hydrophobic photosensitizers, but also allow the oxygen and generated permeability that is essential for PDT [104].

## 8. Conclusions

Surgical excision is still the first choice for treatment of BCC, but when surgery is infeasible due to tumor size or location or patient clinical characteristics or when it is necessary to take into consideration the cosmetic outcome, PDT should be considered as a good treatment option. Differences in photosensitizers, patient preparation, and proper total amount of light irradiation or number of PDT cycles may result in different treatment outcome. However, no significant differences in recurrence rates of PDT-treated BCC were found between immunocompetent or solid organ transplanted patients [105], demonstrating the feasibility of this technique also in those patients in which others therapeutics approach cannot be used.
